# Effect of Various Acid Solutions on the CO_2_ Dissolution Rate, Morphology, and Particle Size of Precipitated Calcium Carbonate Synthesized Using Seashells

**DOI:** 10.3390/ma16247665

**Published:** 2023-12-15

**Authors:** Yu Jeong Yun, Siwoo Lee, Yangdo Kim, Young Bok Ryu

**Affiliations:** 1 Green Materials and Processes R&D Group, Korea Institute of Industrial Technology, Ulsan 44413, Republic of Korea; toto5937@kitech.re.kr (Y.J.Y.);; 2 Department of Materials Science and Engineering, Pusan National University, Busan 46241, Republic of Korea

**Keywords:** seashell, precipitated calcium carbonate, carbonate, acid solution type

## Abstract

In this study, the influence of acid solutions on the production of precipitated calcium carbonate (PCC) using seashells was investigated. In terms of the Ca dissolution efficiency and atmosphere for dissolving CO_3_^2−^, the results indicate that HCl, HNO_3_, CH_3_COOH, and HCOOH at 1.0 M were the most ideal among the acid solutions. The use of weak acids resulted in the low degree of dissolution of Al and Fe. These impurities could be mostly removed through the pH adjustment process, leading to PCC with a purity of 99% or more. Further, CH_3_COOH and HCOOH exhibited low CaCO_3_ carbonation efficiency owing to the hydrogen bonding of the carboxyl group and its hindering effect on the growth of CaCO_3_ particles. In addition, in the presence of the carboxyl group, the morphology tended to be oval, and the particle size was small. Particularly, when CH_3_COOH was used, the combined effect of the low initial Ca ion concentration and slow CO_2_ dissolution rate resulted in minimal changes during the carbonation time and the smallest particle size. However, variations in the degree of Ca concentration with a change in the acid solution concentration influenced the dominance of nucleation and particle growth, leading to variations in the particle size. The results of this study revealed that when manufacturing PCC using seashells, the appropriate acid solution must be selected to obtain the required PCC properties.

## 1. Introduction

With the rapid population growth in the 20th century, the demand for food has increased significantly, leading to substantial growth in the aquaculture industries [1,2]. Particularly, shellfish farming, which accounts for approximately 43% of seafood production, is one of the most important industries in the marine aquaculture field [3]. Along with the increase in the consumption of shellfish, concerns about the disposal of shellfish waste have also increased. Seashells, which make up the majority of shellfish, are sometimes used as fertilizers or handicrafts [4], but most seashells are not utilized and are left unused near production sites [5,6]. Neglected seashell wastes not only damage the landscape but also cause serious public problems, such as the emission of toxic gases, such as NH_3_ and H_2_S, during microbial decomposition [7,8,9]. To solve these problems, various studies related to seashell recycling are being conducted.

Seashell is mainly composed of calcium carbonate (CaCO_3_), which is the same as limestone [3]. In terms of its components and waste recycling, it is highly useful as a substitute for the depleting limestone resources [10,11,12,13]. Generally, limestone is used as ground calcium carbonate (GCC), which is simply produced by grinding/crushing, or precipitated calcium carbonate (PCC), which is produced chemically. Compared to GCC, PCC has a higher purity, making it relatively more expensive. In addition, it can be easily tailored to meet the required characteristics (e.g., grain size and morphology). Accordingly, the demand for PCC has increased recently. Therefore, research is being conducted on the use of seashells as a raw material for PCC production instead of limestone. Ramakrishna et al. [14] produced PCC with a size of 30–40 μm through calcination and hydration processes. In a later study, PCC with an aragonite crystalline phase was produced through a calcination and hydration process, and its feasibility for coffee waste treatment was examined [15]. Yuliatun et al. [12] dissolved calcined seashells using HNO_3_ and NH_3_ and then carbonated them to produce PCC, which is a mixture of calcite and vaterite. However, the high-temperature calcination process of 700 to 900 °C performed in these aforementioned studies requires a high energy consumption for heat treatment, resulting in a higher generation of CO_2_ compared to the amount of CO_2_ consumed during an actual PCC production process [16,17]. In addition, separate mass calcination facilities are required [18], and the removal of impurities during this process is difficult.

Depending on its purity, morphology, crystal size, and crystalline phase, there is a wide application range for PCC. First, purity is an important characteristic in precision chemical industries that utilize PCC, such as paper, plastic, and rubber industries. In the paper or paint industry, the whiteness of PCC affects the final color of the product [19]. Generally, the lower-purity PCC has a lower whiteness, so high-purity PCC is required [20,21]. Additionally, after the removal of impurities, PCC can be used in the pharmaceutical and food fields [22]. Second, the characteristics of the product vary depending on the morphology of PCC. For example, PCC with an elliptical or spherical morphology exhibits superior loading efficiency of biomolecules compared to those with a cubic morphology [23]. Typically, PCCs with a hollow or porous spherical structure are useful for drug delivery and cancer treatment [24,25]. Third, the particle size of PCC is an important factor when PCCs are used as a polymer composite. As the particle size of PCC decreases, the mechanical strength of the composite, such as tensile yield stress and ultimate strength, increases [26,27,28]. Additionally, when PCC with a small particle size is used as the filler in paper, the optical properties of the paper become excellent [29]. Fourth, the crystalline phases of calcium carbonate are largely divided into calcite, aragonite, and vaterite [30]. Aragonite, a metastable phase, is mostly needle-like and columnar and has a high aspect ratio, so it can improve the mechanical properties of paper or polymer materials [31,32]. Vaterite, which has a spherical or ring shape, is an unstable phase characterized by high solubility, small specific gravity, and high dispersion [33,34]. Calcite has the most thermodynamically stable structure at room temperature and pressure and has various crystal forms, such as rhombohedral, cubic, spindle, and chain, and is the most widely used in industries [35,36]. To manufacture PCC with the desired physical properties, the process conditions need to be adjusted depending on the application to achieve the required purity, crystal structure, particle size, and other specifications.

Until recently, PCC has been mainly manufactured from limestone, and there have only been a few cases of applying extraction solutions in the manufacturing process using limestone or shell. However, in experiments based on the use of slag as the raw material, researchers have investigated the effect of extraction solutions on the CaCO_3_ carbonation efficiency and PCC characteristics. Lee et al. [37] compared the effects of ammonium salts (NH_4_NO_3_, NH_4_Cl, CH_3_COONH_4_, and (NH_4_)_2_SO_4_) on PCC production using blast-furnace slag. They observed that among the extraction solutions, (NH_4_)_2_SO_4_ exhibited a relatively low Ca extraction efficiency and carbonation efficiency. In the case of CH_3_COONH_4_, Ca(C_2_H_3_O_2_)_2_ was synthesized in addition to CaCO_3_ (calcite) during the carbonation process. Mun et al. investigated the effect of various acid types on PCC manufacturing using blast-furnace slag and waste cement and confirmed the suitability of HCl, CH_3_COOH, and NH_4_Cl for Ca extraction. Additionally, they confirmed the suitability of HCl and CH_3_COOH for synthesizing calcite and NH_4_Cl for synthesizing flower-shaped vaterite [38]. Jo et al. compared the effects of HCl, CH_3_COOH, and NH_4_Cl on three cementitious materials and found that calcite and vaterite were formed for all three extraction solutions. Additionally, NH_4_Cl exhibited the highest Ca extraction and carbonation efficiency [39]. However, studies based on the use of slag focus primarily on the effect of extraction solutions on the Ca extraction efficiency, carbonation efficiency, and PCC crystal type. Additionally, research on other PCC characteristics, such as morphology and particle size, is limited.

Therefore, in this study, we examined the effect of acid solutions on the Ca dissolution efficiency, CO_2_ dissolution rate, and CaCO_3_ carbonation efficiency and observed changes in the characteristics of the produced CaCO_3_, such as crystalline phase, morphology, and particle size.

## 2. Materials and Methods

### 2.1. Materials

The seashell powder used to manufacture PCC was supplied by Hanreo Chemical, located in Tongyeong City, Republic of Korea. The supplied powder was separated into particle sizes of 200 μm or less and used. To dissolve Ca ions in the seashell powder, HCl (35–37%, Daejung, Siheung, Republic of Korea), HNO_3_ (68–70%, Samchun, Seoul, Republic of Korea), CH_3_COOH (99.5%, Daejung), HCOOH (99%, Samchun), H_2_SO_4_ (95%, Samchun), and H_3_PO_4_ (85%, Samchun) were individually employed as pure acid solutions.

### 2.2. Methods

**Figure 1:**
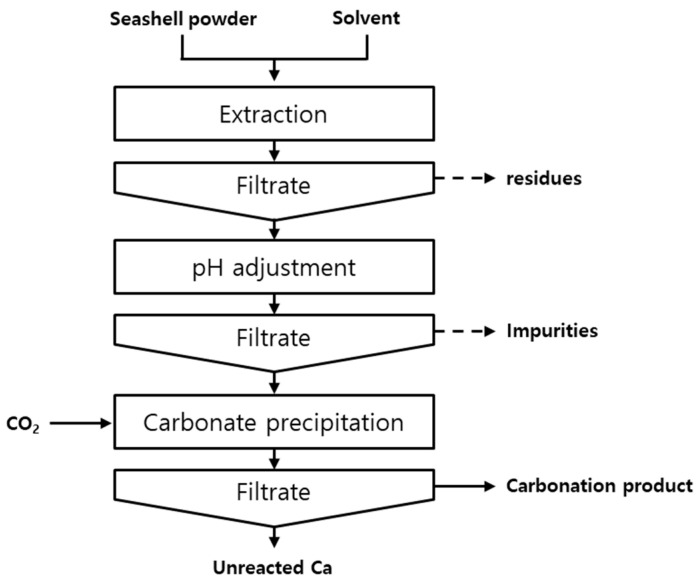
Schematic of the precipitated calcium carbonate synthesis process using seashell powder.

Figure 1 shows the overall schematic of the experimental process. The experiment is divided into three stages: dissolution, pH adjustment, and carbonation.

#### 2.2.1. Dissolution

Seashell powder and various concentrations of the respective acid solution (0.1, 0.5, 1.0, 1.3, 1.5 M) were stirred at 300 rpm for 30 min at a ratio of 5 g/100 mL. After the dissolution process was finished, the Ca-rich solution and residue were separated through a glass filter. The solubility of the seashell powder according to the type and concentration of the acid solution was calculated as follows (Equation (1)).

(1) $$Solubility\left(\%\right)=\frac{W_{s}\left(g\right)-W_{r}(g)}{W_{s}(g)}\times 100$$

where $W_{s}$ is the weight of the seashell powder, and $W_{r}$ is the weight of the residue after dissolution.

#### 2.2.2. pH Adjustment

The separated Ca-rich solution was adjusted to pH 11 using 3 M NaOH to adjust the pH range in which CO_3_^2−^ dissolves. The precipitate generated during the pH adjustment process was separated from the Ca-rich solution through filtration.

#### 2.2.3. Synthesis of PCC

The carbonation reaction was conducted by stirring the mixture at 300 rpm and injecting CO_2_ (99.99%) at 0.3 L/min into the pH-adjusted Ca-rich solution. The pH of all the carbonation experiments was maintained at 10.5–11 using 3 M NaOH. To investigate the effect of the carbonation time, carbonation reactions were performed for 5, 10, 15, 20, and 25 min for 1 M of each of the acid solutions. Thereafter, to examine the PCC production behavior according to the concentration of the acid solution, two types of acid solutions were selected based on the weight of the product, and experiments were performed for each concentration. Ca-rich solutions were prepared at different acid solution concentrations (0.1, 0.5, 1.0, 1.3, and 1.5 M) and carbonated for 10 min. During carbonation, CO_2_ (99.99%) was injected at 0.3 L/min, the stirring speed was maintained at 300 rpm. The pH of the Ca-rich solution was maintained between 10.5 and 11. When the carbonation reaction was completed, the resulting precipitate and solution were separated through filtration. During the filtration process, the precipitate was washed 4 times with DI water. The resulting product was dried in an oven at 100 °C for 24 h and then ground in a mortar and pestle for the subsequent analysis. The CaCO_3_ carbonation efficiency was calculated as follows (Equation (2)):

(2) $$CaCO_{3}\;carbonation\;efficiency\left(\%\right)=\frac{W_{f}\left(g\right)\times \frac{M_{Ca}}{M_{CaCO_{3}}}\times 1000}{C_{Ca}\;\left(\frac{mg}{L}\right)\times \frac{1}{4}}\times 100$$

where $W_{f}$ is the weight of the final product, $M_{Ca}$ and $M_{CaCO_{3}}$ are the molecular weights of Ca and CaCO_3_, respectively, and $C_{Ca}$ is the Ca ion concentration in the Ca-rich solution.

#### 2.2.4. CO_2_ Dissolution Rate

To examine the CO_2_ dissolution rate for each acid solution, NaOH was injected to adjust the pH of each 1 M acid solution to 11 or higher, and CO_2_ (99.99%) was passed into the acid solutions at 0.3 L/min. As CO_2_ was passed into the solution, the pH changes were observed in real time and recorded at 2 s intervals. The CO_2_ injection progressed until the pH no longer decreased. The pH reduction rate from pH 11 to 8 for each acid solution was calculated using the equation below (Equation (3)).

(3) $$\frac{pH}{sec}=-\left(\frac{L_{pH8}-L_{pH11}}{T_{pH8}-T_{pH11}}\right),$$

where $L_{pH8}$ and $L_{pH11}$ are the pH values closest to 8 and 11 among the recorded pH values, and $T_{pH8}$ and $T_{pH11}$ are the times when the pH values are closest to 8 and 11, respectively.

### 2.3. Analysis

The compositions of the raw material and the generated PCC were analyzed using an X-ray fluorescence spectrometer (XRF, SHIMADZU/XRF-1800, Shimadzu Scientific, Kyoto, Japan). To verify the crystal structures of the raw materials and residual substances after leaching, X-ray diffraction analysis was performed using an X-ray diffractometer (XRD, X’pert3-Powder, PANALYTICAL, Sydney, Australia). The crystalline phases and their proportions were analyzed using XRD patterns. The ion concentrations in the Ca-rich solutions were analyzed using an inductively coupled plasma spectrometer (ICP, iCAP6500, THERMO ELECTRON, Waltham, MA, USA). The detection limit for analyzing the ion concentration was 0.5 ppm. After carbonation, the morphology of the product was observed using scanning electron microscopy (SEM, SU8020, Hitachi, Hitachi shi, Japan). Additionally, the particle size and distribution of the products were determined using a particle size analyzer (PSA, LS 13 320, Beckman Coulter, Brea, CA, USA).

## 3. Results

### 3.1. Seashell Analysis

**Figure 2:**
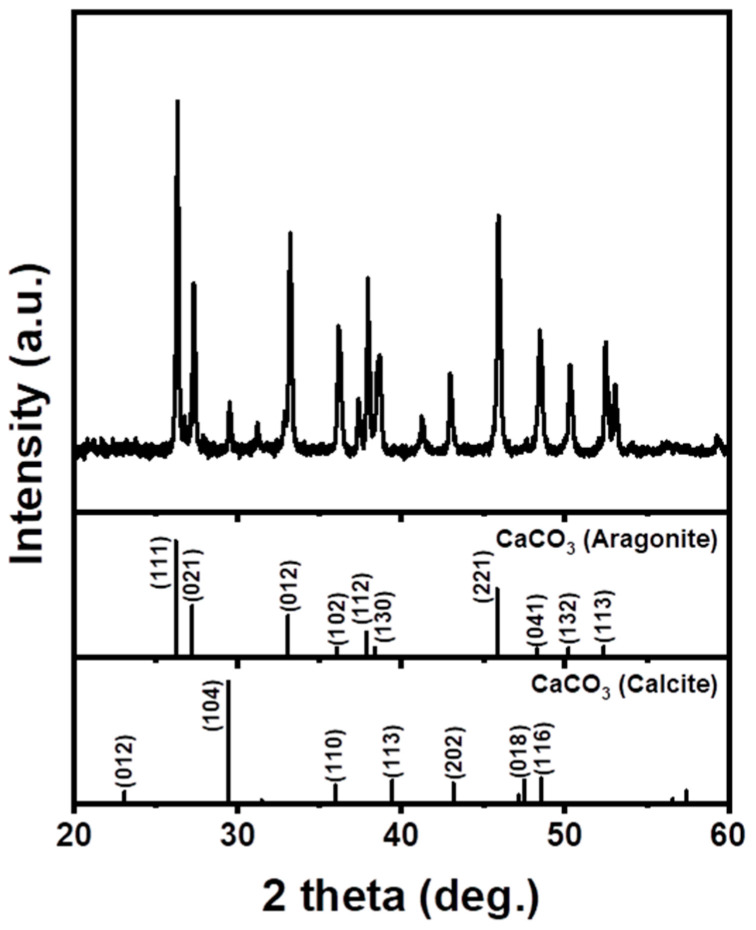
X-ray diffraction (XRD) patterns of the seashell powder.

The results of the XRF analysis of the seashell powder used in this study are summarized in Table 1. The analysis results confirmed that the seashell was composed of approximately 94% Ca. In addition, it also contained Si, Na, and Cl, which are believed to have been introduced through exposure to seawater. The XRD analysis of the seashell powder confirmed the presence of mixed calcite and aragonite at a ratio of approximately 1:15 (Figure 2).

### 3.2. Ca^2+^ Dissolution from Seashell Powders

**Figure 3:**
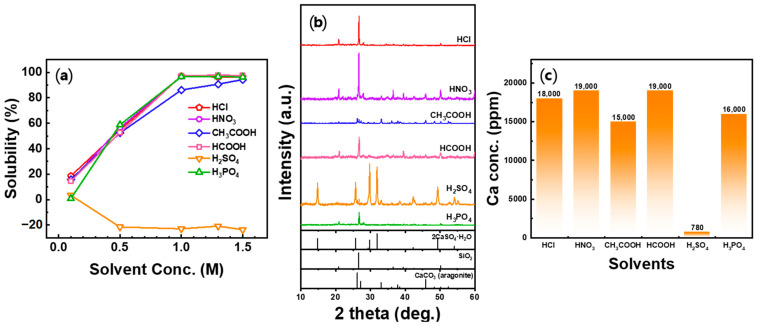
Effect of acid solution on the dissolution from seashell powder: (**a**) solubility, (**b**) XRD patterns of the residue after dissolution, and (**c**) Ca concentration of the Ca-rich solution after dissolution (Solid/liquid ratio: 5 g/100 mL, dissolution time: 30 min, stirrer speed: 300 rpm).

To evaluate the effect of the acid solution on the Ca dissolution efficiency, the concentrations of six acid solutions (HCl, HNO_3_, CH_3_COOH, HCOOH, H_2_SO_4_, and H_3_PO_4_) were adjusted from 0.1 to 1.5 M. The solubility for each condition is shown in Figure 3a. Excluding H_2_SO_4_, the solubility of Ca increased as the concentration of the acid solution increased. The solubility of Ca in HCl, HNO_3_, HCOOH, and H_3_PO_4_ increased to approximately 97% as the concentration of the acid solution increased up to 1.0 M but did not increase further from 1.3 M. In the case of CH_3_COOH, the solubility continued to increase from approximately 86 to 94% as the concentration increased from 1.0 to 1.5 M. When H_2_SO_4_ was used, more reaction products were obtained than the weight of the seashell powder used at all concentrations except 0.1 M. The XRD analysis of the reaction product (Figure 3b) confirmed the formation of CaSO_4_·H_2_O, which was attributed to the rapid reaction between the dissolved Ca ions and SO_4_^2−^ to form the thermodynamically stable CaSO_4_·H_2_O.

The crystal structure of the residue obtained for different 1.0 M acid solutions was analyzed using XRD and shown in Figure 3b. The SiO_2_ crystal structure was confirmed in the XRD profiles of HCl, HNO_3_, HCOOH, and H_3_PO_4_. Combining these results with the previous solubility findings indicates that most of the Ca ions in the seashell were dissolved at a concentration of 1.0 M for HCl, HNO_3_, HCOOH, and H_3_PO_4_. The residue obtained from the CH_3_COOH acid solution was confirmed to be a mixture of SiO_2_ and CaCO_3_ (aragonite), which is consistent with the raw material of seashell powder. This implies that there were undissolved Ca ions in the 1.0 M CH_3_COOH solution. In the H_2_SO_4_ acid solution, Ca ions precipitated in the form of CaSO_4_·H_2_O, making it difficult to utilize Ca ions in the carbonation process, indicating the unsuitability of H_2_SO_4_ as an acid solution.

The ICP analysis results of the acid solution are shown in Figure 3c. The ICP analysis results revealed that the highest Ca ion concentration was observed in HNO_3_ and HCOOH, followed by HCl, H_3_PO_4_, CH_3_COOH, and H_2_SO_4_. Excluding H_2_SO_4_, CH_3_COOH exhibited the lowest Ca ion concentration among the acid solutions. Previous studies have reported the high Ca dissolution efficiency of H_2_SO_4_ compared to other acid solutions, such as HCl, CH_3_COOH, and NH_4_Cl, in conventional experiments using gypsum [40,41,42] or serpentinite [43] as raw materials. In this experiment, it was used to achieve a high Ca dissolution efficiency, but as the result of the experiment on seashells, the Ca dissolution efficiency was extremely low. Therefore, it was excluded from subsequent experiments. In experiments using existing HCl, a gelation phenomenon that adversely affected leachate separation was observed [44,45], but the seashell had a lower Si content than other waste materials, so the gelation phenomenon did not occur even when HCl was used.

### 3.3. pH Adjustment

**Figure 4:**
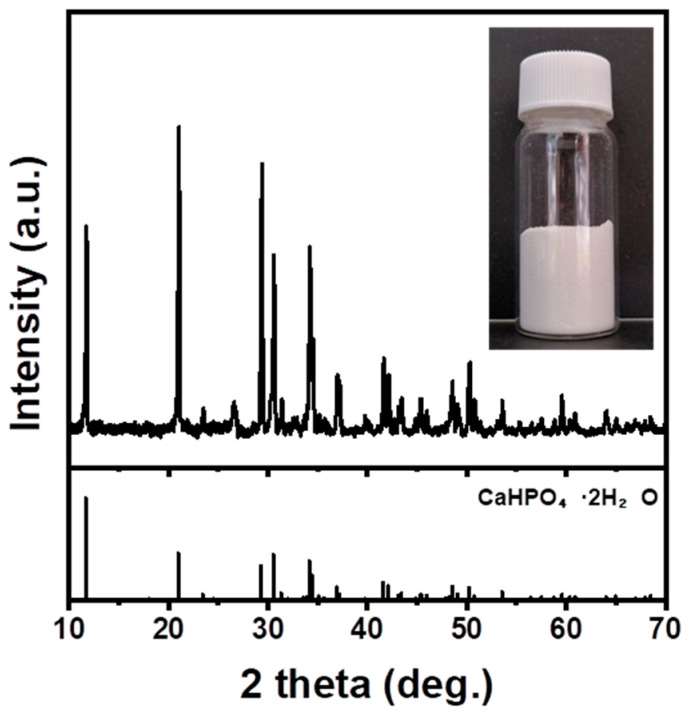
Precipitate produced when H_3_PO_4_ was used during the pH adjustment process.

During the carbonation reaction, the pH should be increased to 11 by adding 3 M NaOH to create an atmosphere for dissolving CO_3_^2−^. During this process, we examined if a change in pH will induce the precipitation of impurities. As NaOH was added to the Ca-rich solution using H_3_PO_4_, a white precipitate was formed, and the pH of the solution remained acidic. The XRD analysis of the precipitate confirmed the formation of CaHPO₄·2H₂O (Figure 4). This indicates the unsuitability of H_3_PO_4_ as an acid solution because of its difficulty in utilizing Ca ions and creating an environment for dissolving CO_3_^2−^.

The pH of four types of the acid solutions, excluding H_3_PO_4_, was adjusted to above 11, and the impurities precipitated during the pH adjustment process were separated. Fe and Al were able to be removed from the seashell through precipitation [46,47]. The removal efficiency of Fe and Al was confirmed through the ICP analysis of the solution before and after pH adjustment (Table 2). The concentrations of Fe and Al ions in the Ca-rich solution prepared using strong acids HCl and HNO_3_ were relatively high. The high acidity of HCl and HNO_3_ dissolved more Fe and Al. Additionally, Fe ions were completely removed through precipitation reaction during the pH adjustment, but Al ions remained at approximately 16 and 4%. The concentrations of the dissolved Fe and Al ions in the weak acids (CH_3_COOH and HCOOH) were relatively low, and it was confirmed that Fe and Al ions could be completely removed through pH adjustment. The XRF analysis results of PCC produced by different acid solutions confirmed the removal of Fe and Al (Table 3). Additionally, PCC produced by HCl and HNO_3_, which were analyzed for Al after pH adjustment, had relatively high Al content in the products, and the purity was approximately 97%. Further, the purity of the product obtained from CH_3_COOH and HCOOH treatments, which showed complete removal of Fe and Al in the ICP analysis, was approximately 99%.

### 3.4. Carbonation

#### 3.4.1. CO_2_ Dissolution Rate

When CO_2_ is dissolved in solution, it undergoes reactions 4, 5, and 6. The dissolution of CO_2_ resulted in a decrease in the pH of the solution owing to the H^+^ generated during this reaction. Therefore, the dissolution rate of CO_2_ in solution can be predicted by comparing the pH reduction rate.

(4) $${CO}_{2}+H_{2}O↔H_{2}{CO}_{3}$$

(5) $$H_{2}{CO}_{3}↔H^{+}+{HCO}_{3}^{-}$$

(6) $${HCO}_{3}^{-}↔H^{+}+{CO}_{3}^{2-}$$

**Figure 5:**
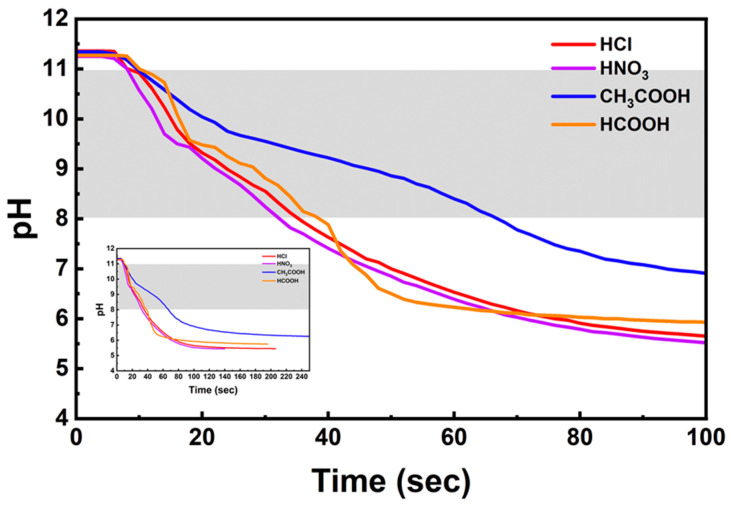
Change in the pH during CO_2_ injection in the absence of Ca ions (Acid solution concentration: 1.0 M, stirrer speed: 300 rpm, pH adjustment: over pH 11 using 3 M NaOH, CO_2_ flow rate: 0.3 L/min).

To examine the influence of the acid solution on the CO_2_ dissolution rate, the pH change after CO_2_ injection into 1 M HCl, HNO_3_, CH_3_COOH, and HCOOH solutions with adjusted pH levels was compared (Figure 5). 

The pH 11–8 range, where the CO_3_^2−^ used to form CaCO_3_ is dissolved, is indicated by the gray part in Figure 5. The degree of pH change per unit time in the corresponding range is shown in Table 4. The fastest CO_2_ dissolution rate was observed in HNO_3_, followed by HCl, HCOOH, and CH_3_COOH. The slowest CO_2_ dissolution rate per unit time was observed in CH_3_COOH, which was approximately half of that of the other acid solutions. The slow dissolution rate of CO_2_ in CH_3_COOH was attributed to the effects of hydrogen bonding and dipole moment. It is known that CH_3_COOH and HCOOH can form hydrogen bonds with water molecules owing to their carboxyl group (-COOH), thus preventing the reaction between CO_2_ and H_2_O. Additionally, the dipole moment of CH_3_COOH is 1.74, which is higher than the dipole moment value of HCOOH (1.41). The strength of hydrogen bonding increases with an increase in the dipole moment. This suggests that the substantial dipole moment of CH_3_COOH results in hydrogen bonding with water molecules, preventing the ionization of CO_2_ molecules and thus slowing down the dissolution rate of CO_2_.

#### 3.4.2. Effect of Carbonation Time

**Figure 6:**
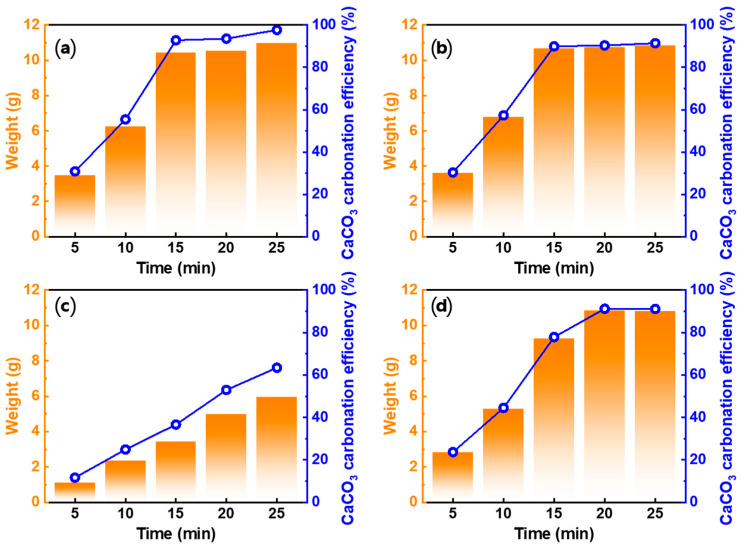
Weight of PCC and CaCO_3_ carbonation efficiency according to the carbonation time using different acid solutions: (**a**) HCl, (**b**) HNO_3_, (**c**) CH_3_COOH, and (**d**) HCOOH (Acid solution concentration: 1.0 M, solid/liquid ratio: 5 g/100 mL, dissolution time: 30 min, stirrer speed: 300 rpm, pH value: pH 10.5–11 using 3 M NaOH, CO_2_ flow rate: 0.3 L/min).

To determine the effect of carbonation time for each acid solution, Ca-rich solutions prepared using 1 M of the acid solutions were carbonated for 5, 10, 15, 20, and 25 min at 0.3 L/min and 300 rpm. The product weight and CaCO_3_ carbonation efficiency over time are shown in Figure 6. With increasing carbonation time, both the product weight and CaCO_3_ carbonation efficiency in all acid solutions increased, but the extent of the increase varied. Although the weight of the product obtained from HNO_3_ was heavier than that obtained from HCl, the CaCO_3_ carbonation efficiency, which was calculated based on the initial Ca ion concentration in each Ca-rich solution, was lower. This was because the initial Ca ion concentration in HNO_3_ was higher than that in HCl. CH_3_COOH has a slower carbonation rate compared to other acid solutions, resulting in the persistence of unreacted Ca ions until 25 min. Further, HNO_3_ exhibited the highest CaCO_3_ carbonation efficiency as a function of the carbonation time, followed by HCl, HCOOH, and CH_3_COOH. These results are consistent with the results from the CO_2_ dissolution rate experiments, indicating that the CaCO_3_ carbonation efficiency was influenced by the variation in the CO_2_ dissolution rates depending on the acid solution type. The product weight of HCOOH, as well as CH_3_COOH, was approximately 1–1.5 g less than that of HCl and HNO_3_. This may be attributed to not only the CO_2_ dissolution rate but also the hindrance of CaCO_3_ growth due to the adsorption of carboxyl groups on CaCO_3_ [48,49,50].

**Figure 7:**
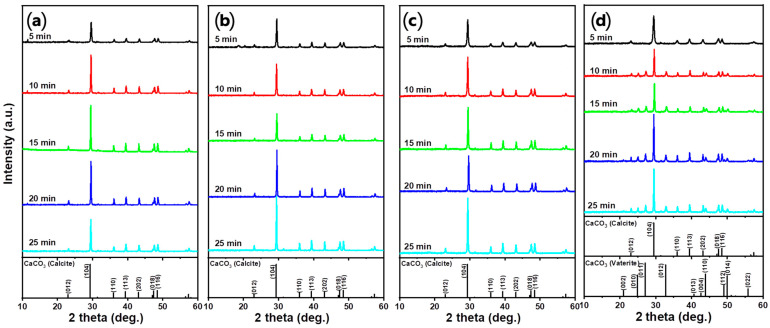
XRD patterns of PCC according to the carbonation time using different acid solutions: (**a**) HCl, (**b**) HNO_3_, (**c**) CH_3_COOH, and (**d**) HCOOH (Acid solution concentration: 1.0 M, solid/liquid ratio: 5 g/100 mL, dissolution time: 30 min, stirrer speed: 300 rpm, pH value: pH 10.5–11 using 3 M NaOH, CO_2_ flow rate: 0.3 L/min).

The XRD analysis results of the precipitated material are shown in Figure 7. Most of the produced PCC was in the form of calcite, which is the most stable crystalline phase at room temperature and pressure. The formation of vaterite was observed in the XRD pattern of HCOOH after 10 min. The formation of vaterite in HCOOH could be attributed to the promotion of the temporary stabilization of vaterite by the carboxyl group [51]. Despite the presence of a carboxyl group in CH_3_COOH, the low initial concentration of Ca ions prevented the formation of vaterite, resulting in the exclusive formation of calcite [52].

**Figure 8:**
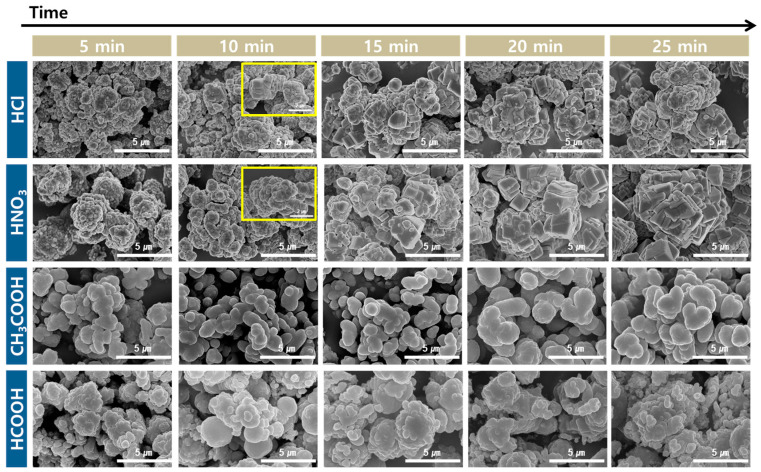
Morphology of PCC according to acid solution and carbonation time (Acid solution concentration: 1.0 M, solid/liquid ratio: 5 g/100 mL, dissolution time: 30 min, stirrer speed: 300 rpm, pH value: pH 10.5–11 using 3 M NaOH, CO_2_ flow rate: 0.3 L/min).

Figure 8 shows the SEM images of the PCC produced according to the carbonation time for different acid solutions. Small PCC spheres, which aggregated at 5 min, were formed when HCl and HNO_3_ were used as the acid solutions. After 10 min, the spherical particles were converted into a rhombohedral shape. The yellow box in Figure 8 shows the morphology of the particle in more detail. From 15 to 25 min, plate-like structures accumulated to form rhombohedral- and polygonal-shaped particles with rough surfaces. When CH_3_COOH was used as the acid solution, oval-shaped particles were formed from 5 to 25 min, and there was only a slight change in the morphology over time. When HCOOH was used as the acid solution, aggregated large spherical and oval-shaped particles were observed, and there was almost no change in the morphology over time. When CH_3_COOH and HCOOH with carboxyl groups were used, the particle morphology was mainly round shape. The growth rate appeared to change owing to the difference in the interaction strength between each crystal plane of CaCO_3_ and the carboxyl group. The carboxyl group (-COOH) dissociated into a -COO- group and then preferentially interacted with the positively charged surface of calcite [53]. Therefore, the interaction strength was stronger in the order of Ca ions charge density per unit square nanometer (104) > (018) > (113), and the growth rate of the specific crystal plane changed. Typically, the dominant growth rate of (104) results in the formation of cubic-shaped calcite. However, the carboxyl group slowed down the growth rate of (104), leading to the formation of oval-shaped particles [54,55,56].

**Figure 9:**
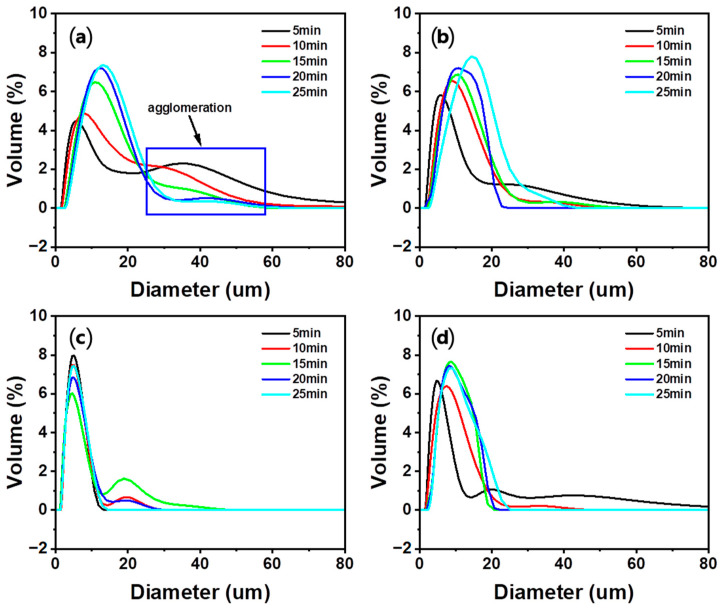
Effect of the carbonation time on the particle size of PCC: (**a**) HCl, (**b**) HNO_3_, (**c**) CH_3_COOH, and (**d**) HCOOH (Acid solution concentration: 1.0 M, solid/liquid ratio: 5 g/100 mL, dissolution time: 30 min, stirrer speed: 300 rpm, pH value: pH 10.5–11 using 3 M NaOH, CO_2_ flow rate: 0.3 L/min).

The results of particle size and distribution analysis are shown in Figure 9 and Table 5. In the Ca-rich solutions obtained using HCl, HNO_3_, and HCOOH, the particle size (D_50_) of the PCC increased as the carbonation time increased. The increase in the particle size with an increase in the carbonation time can be explained by the Ostwald ripening effect. Ostwald ripening is a phenomenon in which small particles dissolve and large particles grow, thus increasing the average particle size [57,58,59]. Moreover, in the particle size distribution result, the agglomeration peak decreased as the carbonation time increased, and it appeared that the weakly bound agglomeration was separated during the carbonation reaction. Compared to the PCC formed in the Ca-rich solution obtained using other acid solutions, there was no significant change in the size of the PCC formed in the Ca-rich solution obtained using CH_3_COOH with a change in the carbonation time. Additionally, a small particle size (approximately 4–5 μm) was maintained in this solution, whereas particles in other acid solutions increased up to 12 µm. The generation of particles with a small size and the maintenance of this size could be attributed to two main reasons. First, CH_3_COOH generates relatively few nuclei because the concentration of Ca ions in this solution is lower than that in other acid solutions. Additionally, because the dissolution rate of CO_2_ is slow, the generation and growth rate of the particles are slow. Second, a carboxyl group is adsorbed on the surface of CaCO_3_ and hinders particle growth [48,49,50]. The effect of the carboxyl group on the particle growth is further confirmed by the smaller particle size of the PCC from HCOOH compared to that from HCl and HNO_3_. Moreover, CH_3_COOH exhibits a slow nucleation rate, and particle growth was hindered, so particle growth during the carbonation process was limited. Therefore, compared to other acid solutions, a small-sized PCC was generated in CH_3_COOH regardless of the particle growth during the reaction time.

#### 3.4.3. Effect of Acid Solution Concentration

**Figure 10:**
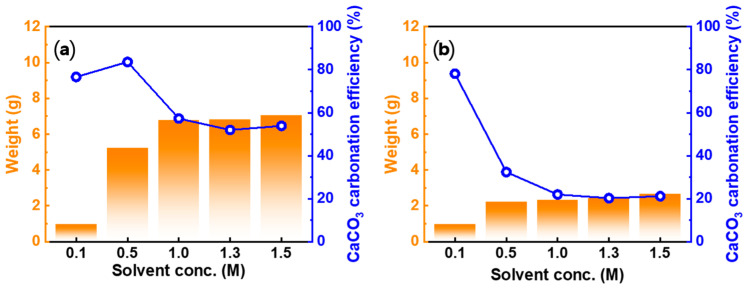
Weight of PCC and CaCO_3_ carbonation efficiency with a change in the acid solution concentration: (**a**) HNO_3_ and (**b**) CH_3_COOH (Solid/liquid ratio: 5 g/100 mL, dissolution time: 30 min, stirrer speed: 300 rpm, pH value: pH 10.5–11 using 3 M NaOH, carbonation time: 10 min, CO_2_ flow rate: 0.3 L/min).

To examine the change in the carbonation behavior as a function of the acid solution concentration, a carbonation experiment was performed using two acid solutions. HNO_3_ and CH_3_COOH were selected because they exhibited the highest and lowest PCC production, respectively, relative to the added seashell in the experiment on the effect of carbonation time. Particularly, CH_3_COOH was selected for further investigation owing to the small particle size of PCC regardless of the reaction time. Figure 10 shows the product weight and CaCO_3_ carbonation efficiency according to acid solution concentration. The weight of the product obtained increased as the concentration of the acid solution increased. In the case of HNO_3_, the CaCO_3_ carbonation efficiency decreased as the concentration of the acid solution increased, except in the range of 0.1 to 0.5 M and 1.3 to 1.5 M. For CH_3_COOH, the CaCO_3_ carbonation efficiency decreased as the concentration of the acid solution increased, except in the range from 1.3 to 1.5 M. The decrease in the CaCO_3_ carbonation efficiency with increasing acid solution concentration was attributed to the higher concentration of Ca ions dissolved in the Ca-rich solution. As the Ca concentration increased, the Ca conversion rate representing the PCC product decreased. Furthermore, the increase in the CaCO_3_ carbonation efficiency was attributed to the fact that the increase in product weight is proportionally larger than the increase in the Ca concentration with an increase in the acid solution concentration.

**Figure 11:**
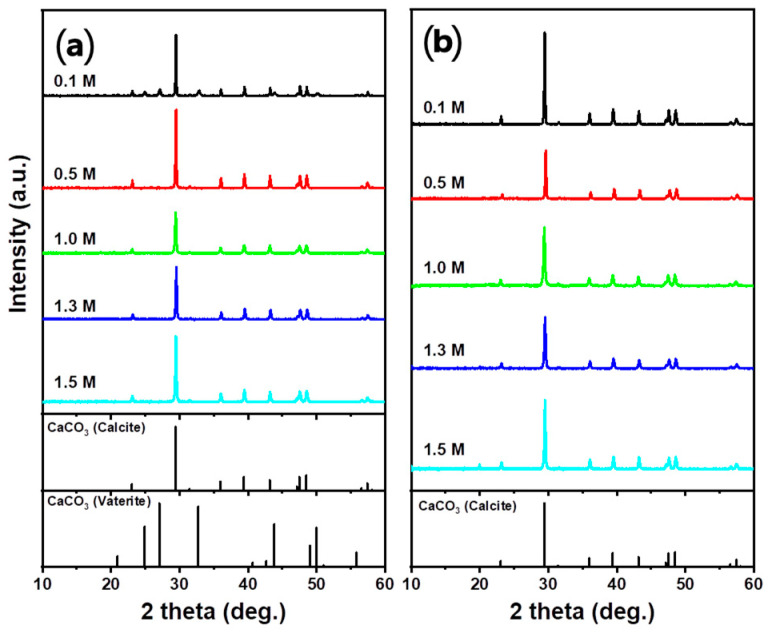
XRD patterns of PCC at different acid solution concentrations: (**a**) HNO_3_ and (**b**) CH_3_COOH (Solid/liquid ratio: 5 g/100 mL, dissolution time: 30 min, stirrer speed: 300 rpm, pH value: pH 10.5–11 using 3 M NaOH, carbonation time: 10 min, CO_2_ flow rate: 0.3 L/min).

The crystalline phase of the PCC was analyzed using XRD analysis (Figure 11). The XRD results confirmed the formation of vaterite in 0.1 M HNO_3_ and the formation of calcite under all other conditions.

**Figure 12:**
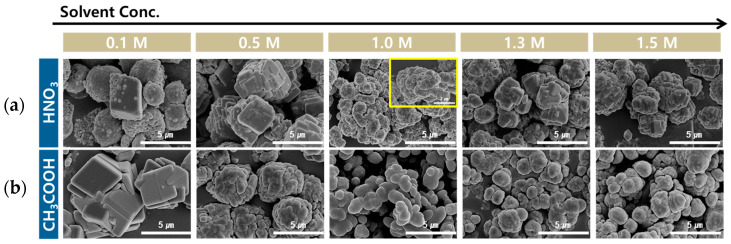
SEM images of PCC according to acid solution concentration: (**a**) HNO_3_ and (**b**) CH_3_COOH (Solid/liquid ratio: 5 g/100 mL, dissolution time: 30 min, stirrer speed: 300 rpm, pH value: pH 10.5–11 using 3 M NaOH, carbonation time: 10 min, CO_2_ flow rate: 0.3 L/min).

The SEM images of the PCC particles obtained at different acid solution concentrations are shown in Figure 12. In the case of HNO_3_, rhombohedral- and spherical-shaped particles with a rough surface were observed simultaneously at 0.1 M. With an increase in the concentrations beyond 0.5 M, plate-like structures accumulated to form rhombohedral- and polyhedral-shaped particles with rough surfaces. The show the particles in more detail, a larger magnification image is indicated in the yellow box. When CH_3_COOH was used as the acid solution, agglomerated rhombohedral particles were observed at 0.1 M. At 0.5 M, oval-shaped particles aggregated into cubic shapes. Above 1.0 M, oval-shaped particles were formed. As the concentration of CH_3_COOH increases, the morphology of PCC gradually becomes more rounded because of an increase in the number of the carboxyl group. Owing to the difference in the interaction strength between the carboxyl group and each crystal plane of CaCO_3_, the growth rate varied for each crystal plane, resulting in a round shape [55,56].

**Figure 13:**
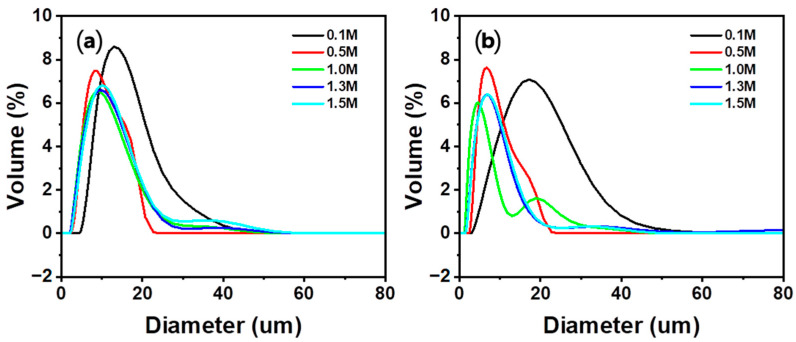
Effect of acid solution concentration on the particle size of PCC: (**a**) HNO_3_ and (**b**) CH_3_COOH (Solid/liquid ratio: 5 g/100 mL, dissolution time: 30 min, stirrer speed: 300 rpm, pH value: pH 10.5–11 using 3 M NaOH, carbonation time: 10 min, CO_2_ flow rate: 0.3 L/min).

The results of particle size analysis are shown in Figure 13 and Table 6. For both acid solutions, as the concentration of the acid solution increased from 0.1 to 1.0 M, the particle size decreased rapidly. However, when the concentration was increased from 1.0 to 1.5 M, the particle size increased. Particle size appears to be affected by the Ca ion concentration in the Ca-rich solution depending on the concentration of the acid solution. According to nucleation and particle growth theory, when the initial Ca ion concentration is low, particle growth dominates nucleation. Thus, with an increase in the concentration of the acid solution, the concentration of Ca ions in the Ca-rich solution increased. This dominance of nucleation over particle growth leads to a reduction in particle size [60,61]. Particularly, the amount of carboxyl groups in CH_3_COOH increased as the concentration of CH_3_COOH increased. Owing to the increased carboxyl group, the inhibition effect on crystal growth becomes stronger, thus increasing the degree of particle size reduction [62]. However, the particle size increased from 1.0 M, which may be attributed to the increased aggregation of particles with an increase in the generation of particles despite the fact that only fine particles are formed [63]. Figure 13 indicates that the size of the agglomerated particles increased with an increase in the concentration from 1.0 to 1.5 M. With an increase in the concentration of the acid solution, the concentration of Ca ions increased, and PCC with small particle size was produced. However, when the concentration of Ca ions exceeded a certain level, the particle size increased owing to the effect of agglomeration. This indicates that to produce PCC with a small particle size, the selected concentration of the acid solution should favor nucleation but hinder agglomeration.

## 4. Conclusions

In this study, the PCC formation behavior as a function of the acid solution was investigated. The results revealed that among the acid solutions examined, HCl, HNO_3_, CH_3_COOH, and HCOOH at concentrations not exceeding 1.0 M exhibited the best Ca dissolution efficiency. Weak acids enabled the production of PCC with a high purity of approximately 99% owing to the low solubility of Fe and Al. Among the four acid solutions, CH_3_COOH exhibited the slowest CO_2_ dissolution rate owing to the influence of hydrogen bonding with water molecules and higher dipole moment. As a result of carbonation experiments using different acid solvents, the weight of the produced CaCO_3_ increased when the carbonation time or the acid solution concentration increased. However, if the degree of increase in Ca ion concentration due to an increase in the acid solution concentration was larger than the increase in the weight of PCC, the CaCO_3_ carbonation efficiency decreased. The morphology of the produced CaCO_3_ was cubic or round-shaped, depending on the presence or absence of the carboxyl group. Generally, the particle size of CaCO_3_ increased as the carbonation time increased because of particle growth. The use of CH_3_COOH resulted in the generation of PCC with a small particle size regardless of the carbonation time, owing to the impediment effect of the carboxyl group on the particle growth and the slow generation and growth of particles. However, when the acid solution concentration was increased beyond 1.3 M, the particle size increased owing to the phenomenon of agglomeration.

When manufacturing PCC using seashells as the raw material, it is important to select the acid solution according to characteristics such as desired purity, particle size, and morphology. When a high purity is required, CH_3_COOH and HCOOH are advantageous, whereas when a small particle size is required, CH_3_COOH is ideal. When higher production is more important than purity and particle size, HCl and HNO_3_ should be selected. Generally, the unit price of PCC with small particle size and high purity is high, so using CH_3_COOH is advantageous for producing high-cost PCC.

## Figures and Tables

**Table 1:** XRF analysis of the seashell powder.

	Ca	Si	Na	Al	Fe	Cl	Etc.
Seashell powder	94.41	1.86	0.86	0.85	0.61	0.53	0.88

**Table 2:** Change in the ion (Fe, Al) concentration of the Ca-rich solution produced with different acid solutions according to pH adjustment (Acid solution concentration: 1.0 M, solid/liquid ratio: 5 g/100 mL, dissolution time: 30 min, stirrer speed: 300 rpm, pH adjustment: over pH 11 using 3 M NaOH).

	HCl		HNO_3_		HCOOH		CH_3_COOH	
	Fe	Al	Fe	Al	Fe	Al	Fe	Al
Before	5.48	5.22	8	12	0.95	3.5	-	1.9
After (pH 11)	-	0.83	-	0.53	-	-	-	-

**Table 3:** Chemical content of PCC according to the XRF results (Acid solution concentration: 1.0 M, solid/liquid ratio: 5 g/100 mL, dissolution time: 30 min, stirrer speed: 300 rpm, pH adjustment: over pH 11 using 3 M NaOH, carbonation time: 10 min, CO_2_ flow rate: 0.3 L/min).

	HCl	HNO_3_	CH_3_COOH	HCOOH
Ca	97.86	97.43	99.26	99.04
Si	0.09	0.06	0.08	0.09
Na	-	-	-	-
Al	1.72	2.23	0.27	0.64
Fe	-	-	-	-
Sr	-	0.12	0.16	0.12
Cl	0.12	-	-	-
etc.	0.21	0.16	0.23	0.10

**Table 4:** pH reduction rate per second from pH 11 to 8, depending on the acid solution (Acid solution concentration: 1.0 M, stirrer speed: 300 rpm, pH adjustment: over pH 11 using 3 M NaOH, CO_2_ flow rate: 0.3 L/min).

	pH/s
HCl	0.113
HNO_3_	0.122
CH_3_COOH	0.052
HCOOH	0.107

**Table 5:** Particle size (D_50_) of PCC according to carbonation time using different acid solutions (Acid solution concentration: 1.0 M, solid/liquid ratio: 5 g/100 mL, dissolution time: 30 min, stirrer speed: 300 rpm, pH value: pH 10.5–11 using 3 M NaOH, CO_2_ flow rate: 0.3 L/min).

	HCl	HNO_3_	CH_3_COOH	HCOOH
5 min	8.31	6.67	4.93	5.59
10 min	9.46	9.06	5.06	7.23
15 min	11.11	9.96	4.91	8.64
20 min	11.71	10.12	5.03	8.74
25 min	12.41	12.95	4.98	9.02

**Table 6:** Particle size (D_50_) of PCC according to acid solution concentration (Solid/liquid ratio: 5 g/100 mL, dissolution time: 30 min, stirrer speed: 300 rpm, pH value: pH 10.5–11 using 3 M NaOH, carbonation time: 10 min, CO_2_ flow rate: 0.3 L/min).

	HNO_3_	CH_3_COOH
0.1 M	13.93	15.99
0.5 M	9.13	7.64
1.0 M	9.06	5.06
1.3 M	9.43	6.70
1.5 M	9.96	6.80

## Data Availability

The data used this study are available upon request from the corresponding author.
